# Transforaminal intradiscal steroid injection versus transforaminal epidural steroid injection for chronic discogenic low back pain with active discopathy: a randomized controlled trial protocol

**DOI:** 10.3389/fmed.2026.1765174

**Published:** 2026-03-24

**Authors:** Jipeng Song, Shijie Liu, Wancheng Lin, Siyuan Yao, Yao Zhang, Meng Yi, Zhengning Luo, Lixiang Ding

**Affiliations:** Department of Spinal Surgery, Beijing Shijitan Hospital, Capital Medical University, Beijing, China

**Keywords:** active discopathy, discogenic low back pain, randomized controlled trial protocol, transforaminal epidural steroid injection, transforaminal intradiscal steroid injection

## Abstract

**Introduction:**

Chronic discogenic low back pain (DLBP) with active discopathy (Modic type 1 changes) is a specific and debilitating phenotype. Transforaminal epidural steroid injection (TESI) and transforaminal intradiscal steroid injection (TISI) are commonly used treatments, yet their comparative efficacy remains uncertain due to a lack of high-quality, direct comparative studies.

**Objectives:**

To compare the clinical efficacy and safety of TISI versus TESI and to investigate whether the anatomical target of corticosteroid delivery (intradiscal vs. epidural) influences clinical and radiological outcomes in patients with chronic DLBP with active discopathy.

**Trial design:**

This is a single-center, prospective, parallel-group, assessor- and patient-blinded, randomized, controlled trial.

**Methods:**

A total of 118 eligible participants will be randomly allocated in a 1:1 ratio to receive either TISI or TESI. The primary outcome is the change in low back pain intensity from baseline to 1-month post-intervention, measured by the Numerical Rating Scale (NRS). Secondary outcomes, assessed at multiple time points up to 12 months, include longitudinal pain intensity (NRS), functional status (Oswestry Disability Index, Japanese Orthopaedic Association score), health-related quality of life (12-item Short Form Health Survey), psychological status (Hospital Anxiety and Depression Scale), radiological changes (Modic classification, intervertebral disc heigh and Pfirrmann grade), and the incidence of procedure-related adverse events. The primary analysis will follow the modified intention-to-treat principle, with longitudinal data analyzed using a linear mixed model.

**Discussion:**

This trial addresses a critical evidence gap by conducting a direct head-to-head comparison of two mechanistically distinct injection therapies. The findings are anticipated to significantly inform clinical practice and guide the development of evidence-based treatment algorithms for managing chronic low back pain with active discopathy.

**Ethics and dissemination:**

The study protocol has been approved by the Ethics Committee of Beijing Shijitan Hospital, Capital Medical University (IIT2024-032-003). Written informed consent will be obtained from all participants. The results of this trial will be submitted for publication in peer-reviewed journals and presented at scientific conferences, regardless of the outcome.

**Clinical trial registration:**

ChiCTR2500096006, https://www.chictr.org.cn/showproj.html?proj=249912.

## 1 Introduction

Low back pain (LBP) is a predominant musculoskeletal disorder and a leading cause of disability worldwide (1, 2). Although acute and subacute episodes often improve within the first 6 weeks, persistent LBP tends to show limited spontaneous improvement over time (3). Chronic LBP imposes a substantial burden at both the individual level, due to pain and functional impairment, and the societal level, through increased healthcare utilization and economic costs (4–6).

Discogenic low back pain (DLBP), primarily attributed to intervertebral disc degeneration, is a major contributor to chronic LBP (7). A specific and clinically relevant phenotype of DLBP is “active discopathy,” often identified by the presence of Modic type 1 changes on magnetic resonance imaging (MRI) (8, 9). These changes, characterized by bone marrow edema and inflammation in the vertebral endplates adjacent to a degenerated disc, are strongly associated with inflammatory-like pain patterns and a poor prognosis (10–13). The local inflammatory milieu within the disc and endplates is now recognized as a key driver of pain in active discopathy, distinguishing it from other forms of degenerative disc disease (8, 14, 15).

The management of chronic DLBP with active discopathy remains challenging after first-line conservative treatments fail. Minimally invasive spinal injections are commonly employed in this context. Transforaminal epidural steroid injection (TESI) is a widely used technique that delivers corticosteroids to the epidural space near the disc and nerve root, aiming to reduce local inflammation (16, 17). The long-term efficacy of TESI for purely DLBP is debated (17–19). Nevertheless, TESI represents a pragmatic “usual-care” active comparator because it remains frequently used in routine practice for chronic low back pain and suspected discogenic presentations, as reflected in comprehensive evidence-based guidelines from the American Society of Interventional Pain Physicians (ASIPP) (16). Importantly, there is genuine clinical equipoise regarding the optimal anatomical target for steroid delivery in active discopathy. Prior clinical data suggest that a strictly defined cohort with discogenic axial low back pain without radiculopathy demonstrated improvements in pain and physical function after fluoroscopically guided TESI, supporting the plausibility of benefit in carefully selected patients (20). Although complications are uncommon and typically minor (e.g., post-procedural pain exacerbation or headache) without causing permanent impairment (21), rare but catastrophic neurologic injuries and even death have been reported, particularly following injections with particulate steroids (22, 23).

An alternative approach that directly targets the presumed source of inflammation is transforaminal intradiscal steroid injection (TISI). By injecting corticosteroids directly into the diseased disc, TISI aims to suppress the local inflammatory cascade within the disc and adjacent endplates (24). Evidence from several studies suggests that intradiscal steroids can provide significant short-term pain relief in DLBP patients, especially those with active discopathy (9, 25, 26). However, beyond the risks shared with other spinal steroid injections, such as infection, nerve injury, and drug allergy, TISI raises specific concerns regarding potential harm from the disc puncture itself, which may theoretically accelerate disc degeneration (27, 28).

While both TESI and TISI are routinely used in the management of chronic DLBP with active discopathy, they differ fundamentally in their anatomical target and technique. High-quality, head-to-head comparative studies are lacking. This evidence gap creates uncertainty for clinicians when selecting the most appropriate interventional strategy. This single-center, prospective, randomized, parallel-group, assessor- and patient-blinded, controlled trial is therefore designed to directly compare the efficacy and safety of TISI versus TESI, and to investigate whether the anatomical target of corticosteroid delivery (intradiscal vs. epidural) influences clinical and radiological outcomes in patients with chronic DLBP with active discopathy.

## 2 Methods and analysis

### 2.1 Study design

**Figure 1:**
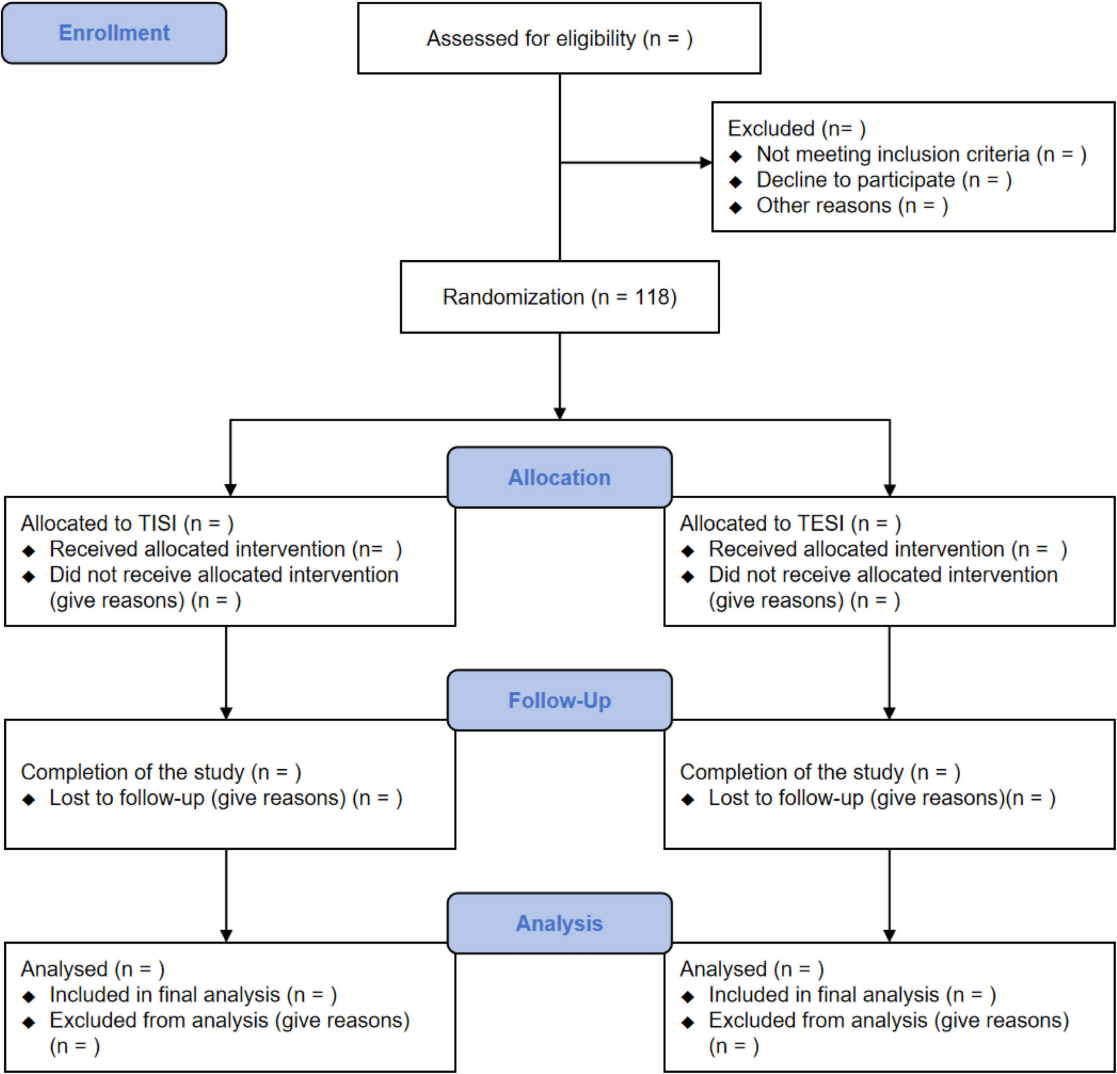
Flow chart of the study.

This study is a single-center, prospective, randomized, parallel-group, assessor- and patient-blinded, controlled trial with a 1:1 allocation ratio. Its primary objective is to compare the clinical efficacy and safety of TISI versus TESI for chronic low back pain associated with active discopathy. This study protocol has been developed and is reported in accordance with the Standard Protocol Items: Recommendations for Interventional Trials (SPIRIT) guidelines (29). The trial schema, including enrollment, intervention, and assessments, is summarized in Figure 1.

### 2.2 Participant eligibility and recruitment

Participant recruitment will be conducted at the Department of Spinal Surgery of Beijing Shijitan Hospital, Capital Medical University, a tertiary care academic center. The recruitment period is scheduled from June 1, 2025, to May 31, 2026. This timeline reflects a protocol amendment implemented after the initial recruitment attempt, as detailed below.

Recruitment was initially planned to begin in February 2025 in accordance with the original trial registration. The first participant was enrolled under that initial protocol; however, during the TISI procedure, intraoperative contrast injection revealed unexpected epidural leakage, indicating the presence of an undetected full-thickness annular fissure. At that time, pre-procedural MRI review did not routinely assess for such fissures, and this criterion was not yet included in the eligibility checklist. This finding prompted a protocol amendment (version 1.4, March 14, 2025) that added MRI evidence of a full-thickness annular fissure as an explicit exclusion criterion and mandated review of annular integrity before enrollment. After ethics approval, formal recruitment was restarted on June 1, 2025, under the revised criteria. To compensate for the interruption, the recruitment period was extended to May 31, 2026. The participant enrolled under the original protocol is not included in the primary analysis cohort and will be reported separately as a protocol deviation case.

During this period, potential participants will be identified from outpatient clinics and the inpatient department. Eligible patients will be fully informed about the study purpose, procedures, potential risks and benefits, and alternative treatments. Written informed consent will be obtained from all participants prior to any study-specific procedures. The inclusion and exclusion criteria are summarized in Table 1.

**Table 1:** The inclusion and exclusion criteria.

Inclusion criteria	Exclusion criteria
(1) Aged between 18 and 70 years. (2) Chronic low back pain persisting for more than 3 months. (3) Pain intensity score of >4 on the 11-point Numerical Rating Scale (NRS), assessed as the average pain level over the preceding week. (4) Failure of a structured, multimodal conservative treatment program (including, but not limited to, physical therapy and non-steroidal anti-inflammatory drugs) for at least 3 months. (5) A clinical diagnosis of DLBP, established in strict accordance with the diagnostic criteria adapted from current expert proposals (37) and the International Society for the Advancement of Spine Surgery (ISASS) recommendations for ICD-10-CM codes (38). This requires meeting all of the following clinical sub-criteria: • Symptom profile: axial midline low back pain with 30 min sitting intolerance, pain aggravated by flexion, and pain that is often relieved by recumbency. The patient may report referred leg pain, but it must be characterized as non-radicular and non-sciatic in nature. • Physical examination: reproduction of concordant low back pain with positive provocation (e.g., sustained passive hip flexion), and the absence of radiculopathy (no motor, sensory, or reflex changes in the lower extremities). • Imaging assessment: radiological evidence of intervertebral disc degeneration from L3 to S1 on a lumbar spine MRI performed within the last 3 months, characterized by one or more of the following: loss of disc signal intensity on T2-weighted imaging, disc height reduction, annular fissure (e.g., high-intensity zone), or loss of normal disc contour. (6) Presence of active discopathy at the same level, defined by Modic Type 1 changes (T1-weighted hypointense, T2-weighted/STIR hyperintense signal) in the adjacent vertebral endplates.	(1) Coexisting severe spinal pathologies or alternative pain sources, including but not limited to: spinal deformity, trauma, fracture, lumbar spinal stenosis, spondylolisthesis (>Grade I), any disc herniation resulting in significant nerve root compression or canal compromise, spinal tumor or infection, specific inflammatory spinal diseases (e.g., ankylosing spondylitis), lumbar myofascial pain syndrome, sacroiliac arthritis, or facet joint syndrome. (2) MRI evidence of a full-thickness annular fissure, which would predispose to injectate leakage and confound the treatment assignment. (3) Modic type 1 changes present at multiple lumbar levels. (4) History of previous lumbar spine surgery. (5) Any contraindication to spinal injection procedures (e.g., known allergy to corticosteroids or iodinated contrast media; systemic infection or local skin infection at the planned puncture site; severe coagulopathy). (6) Pregnancy or lactation. (7) Psychiatric disorders with inability to cooperate. (8) Refusal to provide informed consent, or inability/unwillingness to complete the required follow-up assessments.

Notably, provocation discography will not be used as a diagnostic criterion in this trial. This decision is based on several factors: First, the procedure itself is controversial due to concerns that disc puncture may accelerate degenerative changes (27, 28). Second, as this trial aims to evaluate the specific effect of TISI versus TESI, performing discography in the TESI group would introduce a confounding variable (a prior disc puncture) that could independently influence outcomes and thus invalidate a direct comparison of the two techniques. Therefore, the diagnosis will rely on a non-invasive yet rigorously defined combination of clinical and standard MRI criteria as outlined above.

To characterize the study population and to assess the comparability of the two treatment groups, a comprehensive set of baseline variables will be collected for all participants prior to randomization. These variables include demographic characteristics such as age, sex, body mass index (BMI), education level, employment status, smoking status, and alcohol consumption; clinical characteristics including duration of low back pain (months), pain intensity measured by the Numerical Rating Scale (NRS) at rest and during activity, functional disability assessed by the Oswestry Disability Index (ODI) and Japanese Orthopaedic Association (JOA) score, health-related quality of life measured by the Physical Component Summary and Mental Component Summary of the 12-item Short Form Health Survey (SF-12), and psychological status evaluated by the Hospital Anxiety and Depression Scale (HADS); medication history encompassing current and prior use of analgesics (including nonsteroidal anti-inflammatory drugs (NSAIDs), acetaminophen, and opioids), muscle relaxants, and any previous spinal injections; radiological characteristics including Modic type (confirmed as type 1 for inclusion), Pfirrmann grade, and intervertebral disc height at the target level, as assessed by lumbar spine MRI performed within 1 week prior to enrollment; and prior treatments such as history of physical therapy, chiropractic care, acupuncture, or other conservative treatments, with documentation of treatment duration and response. All baseline data will be collected by trained research personnel using standardized case report forms. These variables will be used to describe the demographic and clinical profile of the study cohort, verify the success of randomization by comparing baseline characteristics between groups, and, if necessary, adjust for potential confounders in the primary and secondary analyses using linear mixed models.

### 2.3 Interventions

#### 2.3.1 Treatment procedure

All injection procedures will be performed by a single spine surgeon with over 10 years of experience. Procedures will be conducted under strict aseptic conditions and with continuous fluoroscopic guidance. Antibiotic prophylaxis will not be administered, consistent with current standard practice for spinal injections.

All procedures will be performed via a transforaminal approach. For TISI, the needle will be advanced into the center of the nucleus pulposus. Intradiscal placement will be confirmed with approximately 0.5 mL of contrast medium (Iohexol). The contrast dispersion pattern will be evaluated to rule out full-thickness annular fissure. A contained, globular pattern will confirm a “successful TISI.” Significant epidural leakage of contrast, indicating a full-thickness annular defect, will be recorded as a “technical failure.” Following confirmation, a 2 mL therapeutic mixture containing 5 mg (1 mL) Betamethasone and 1 mL of 1% lidocaine will be slowly injected into the disc. For TESI, the needle will be positioned in the posterior epidural space. Epidural placement will be confirmed by observing appropriate contrast flow following injection of 0.5 mL of contrast medium (Iohexol). Once correct placement is verified, a 6 mL therapeutic mixture containing 5 mg (1 mL) Betamethasone and 5 mL of 1% lidocaine will be injected. After the procedure, patients will be required to remain in bed for at least 2 h and will be under observation in the ward.

#### 2.3.2 Rescue therapy protocol

To safeguard participant welfare, a standardized protocol for managing acute exacerbation of LBP during follow-up is established. An exacerbation is defined as an increase in the Numerical Rating Scale (NRS) score for LBP to ≥7, sustained for more than 48 h, and/or a significant deterioration in function that prompts the participant to seek urgent care. Upon reporting such pain, a prompt clinical and radiological (MRI) evaluation will be performed to rule out serious complications (e.g., new disc herniation, infection). Before administering any rescue therapy, the current pain intensity and functional status will be recorded as the new baseline for that episode. A stepwise rescue treatment pathway is predefined:

Step 1 (non-invasive): short-term oral analgesics (e.g., NSAIDs or weak opioids) for a predefined maximum duration of 14 days.
Step 2 (invasive): if Step 1 fails, an interlaminar epidural steroid injection at the same spinal level is permitted.
Step 3 (alternative interventions or surgery): if pain remains severe and functionally disabling after Steps 1 and 2, alternative therapeutic options will be evaluated. These may include other interventional procedures (e.g., methylene blue injection or intradiscal thermal procedures) or, as a last resort following confirmation of failure of comprehensive non-surgical management, evaluation for surgical procedures (e.g., minimally invasive or open lumbar fusion).

All rescue therapies will be meticulously documented. The receipt of any additional steroid injection (Step 2) or any invasive procedure (Step 3) for the target DLBP will be considered a major protocol deviation. The use of Step 1 oral analgesics will be recorded as a minor protocol deviation.

### 2.4 Outcomes

#### 2.4.1 Primary outcome

The primary outcome is the efficacy of the intervention, measured as the change in low back pain intensity from baseline to 1-month post-intervention using the NRS (range 0–10, from “no pain” to “worst pain imaginable”). The 1-month primary endpoint was pre-specified to capture the early/peak pharmacodynamic response to corticosteroid injection, which is commonly evaluated at approximately 4 weeks in injection trials. This choice aligns with established precedents in the field: randomized controlled trials of intradiscal glucocorticoid injection in active discopathy have used a 1-month primary endpoint (9, 26), and a high-quality randomized trial of epidural steroid delivery have similarly prespecified 4-week outcomes as primary efficacy assessments (30). Adopting the same design allows direct comparability and meta-analytic synthesis, strengthening the evidence base.

Importantly, this short-term primary endpoint is not intended to represent the ultimate clinical value of treatment for a chronic condition. Consistent with the ICH E9 (R1) estimand framework, the 1-month comparison serves as a confirmatory hypothesis-testing anchor for the immediate treatment effect, while durability will be evaluated through prespecified follow-up at 3, 6, and 12 months. The longitudinal trajectory of NRS will be analyzed using a linear mixed model, with 12 months considered a key time point for sustained efficacy assessment.

#### 2.4.2 Secondary outcomes

Secondary outcomes encompass multiple domains to comprehensively evaluate the clinical efficacy and safety of the interventions over time. Pain intensity, assessed with the NRS, will be evaluated up to 12 months post-intervention to examine the sustainability of treatment effects beyond the primary endpoint. Functional outcomes will be measured using the JOA score (range: 0–29; higher scores indicating better function) and the ODI (range: 0–100%; higher scores indicating greater disability). Health-related quality of life will be evaluated with the Physical Component Summary and Mental Component Summary of the 12-item Short Form Health Survey (SF-12), both ranging from 0 (worst) to 100 (best). Psychological status will be assessed using the Hospital Anxiety and Depression Scale (HADS), with subscale scores ranging from 0 (no clinically significant symptoms) to 21 (maximum clinically significant symptoms). Radiological outcomes, evaluated by two independent blinded radiologists using lumbar spine MRI at 6 and 12 months, will include changes in Modic classification and intervertebral disc height. To provide complementary information on the severity of disc degeneration, we also added Pfirrmann grade as an exploratory radiological outcome. This addition was made prior to the start of patient recruitment (protocol version 1.4, March 14, 2025) and was approved by the Ethics Committee. All radiological assessments will be performed with the assessors blinded to group allocation and clinical outcomes. Safety outcomes will include the incidence of all procedure-related adverse events, categorized as minor (e.g., post-procedural pain, headache, bleeding, infection) or serious (e.g., nerve injury, allergic reactions, paralysis, death).

All outcome data will be collected by blinded assessors using standardized case report forms during scheduled clinic visits at 1, 3, 6, and 12 months. Additional safety and pain assessments will be conducted immediately post-procedure and at 48 h, which may be performed via telephone interview.

### 2.5 Sample size

The sample size calculation was based on the primary outcome of the change in low back pain intensity measured by the NRS at 1-month post-intervention. This time point was pre-specified as the confirmatory endpoint and provides a pragmatic anchor for sample size estimation; however, the longitudinal analysis will incorporate all repeated measures through 12 months to characterize the full time course and durability of effects. According to previous literature (31, 32), the standard deviation of NRS scores was assumed to be 2.6. The minimal clinically important difference (MCID) for NRS was set at 1.65 points (33). With a two-sided significance level of *α* = 0.05 and 90% power, a sample of 53 participants per group is required. Allowing for a 10% dropout rate, the total sample size has been increased to 59 participants per group, resulting in a total of 118 participants.

### 2.6 Randomization and blinding

#### 2.6.1 Randomization

Eligible participants who provide written informed consent will be randomly assigned in a 1:1 ratio to either the TISI group or the TESI group. The randomization sequence will be computer-generated by an independent statistician who is not involved in participant recruitment or clinical management.

#### 2.6.2 Allocation concealment

Allocation concealment will be implemented using sequentially numbered, opaque, sealed envelopes. The assigned treatment for each participant will be placed in individual envelopes by an independent research assistant not involved in the trial. The operating surgeon will open the envelope only after the participant has been formally enrolled in the trial and immediately before the injection procedure, thus ensuring the allocation is concealed until the point of intervention.

#### 2.6.3 Blinding

Given the inherent technical differences between transforaminal intradiscal steroid injection (TISI) and transforaminal epidural steroid injection (TESI), the operating surgeon performing the injections cannot be blinded to group allocation. However, rigorous measures are implemented to maintain patient blinding and outcome assessor blinding.

Participants will not be informed of their specific group assignment. To ensure that both procedures are presented identically from the patient’s perspective, all injections are performed with the patient in the prone position under standard surgical draping that completely obscures the patient’s view of the fluoroscopy screen, the surgeon’s hands, and the syringe. The verbal instructions and descriptions given during the procedure are standardized and neutral (e.g., “we are now administering the medication” or “you may experience some discomfort”), without any reference to the target tissue, the volume being injected, or the contrast distribution pattern. Both groups receive the same medications (betamethasone and lidocaine) with contrast agent, differing only in total volume (2 mL in TISI vs. 6 mL in TESI)—a difference unlikely to be perceived by the patient under draping. Post-procedure care and instructions are identical for both groups, and the overall procedure duration is comparable. To evaluate the success of patient masking, a brief blinding index questionnaire will be administered immediately after the procedure. Patient responses will be used to calculate a Bang’s Blinding Index (BBI) (34) to quantify the degree of unblinding. If the blinding assessment reveals substantial unblinding (e.g., |BBI| > 0.2 in either arm), a sensitivity analysis will be performed excluding patients with correct guesses to assess the robustness of the primary findings.

All research personnel responsible for collecting outcome data (including NRS, ODI, JOA, SF-12, HADS scores, and imaging assessment) during follow-up visits will be blinded to group allocation. These assessors will not be present during the injection procedures and will have no access to operative notes, fluoroscopy images, or medical records that might reveal the treatment assignment. The statistician performing the final data analysis will be blinded to group identity; the two groups will be labeled with non-identifiable codes (e.g., Group A and Group B) until the primary analysis is complete.

### 2.7 Data collection and management

#### 2.7.1 Data collection

All outcome data will be collected by blinded assessors using standardized case report forms during scheduled clinic visits. Patient-reported outcomes (NRS, ODI, JOA, SF-12, HADS) will be assessed using validated Chinese versions of these instruments. To ensure data quality, all research staff will receive standardized training in assessment procedures, and source data verification will be performed. Strategies to promote participant retention include flexible scheduling of follow-up visits, reminder systems (telephone/WeChat software), and compensation for transportation costs.

#### 2.7.2 Data management

Electronic data entry will utilize a secure web-based system with built-in range checks. All data will be doubly verified by independent researchers. De-identified research data will be stored on password-protected institutional servers with regular backups, accessible only to authorized personnel. Paper records will be maintained in locked cabinets.

### 2.8 Statistical methods

#### 2.8.1 Analysis sets

The primary analysis will follow a modified intention-to-treat principle, analyzing all randomized participants who received the allocated intervention and have at least one post-baseline assessment, regardless of subsequent protocol deviations or rescue therapies. A supporting per-protocol analysis will exclude participants with major protocol violations. Predefined violations include: (1) violation of key eligibility criteria; (2) receipt of the incorrect allocated intervention; (3) receipt of any additional invasive spinal procedure (as defined in the rescue therapy protocol) for the target discogenic low back pain during the trial period; and (4) for the TISI group, intraoperative observation of epidural contrast leakage, indicating a full-thickness annular fissure and representing a technical failure to achieve contained intradiscal delivery. To explore the mechanistic role of annular integrity, a supplementary as-treated analysis will compare outcomes among three groups: successful TISI (contained injection), TISI with epidural leakage, and TESI.

As described in the Participant Eligibility and Recruitment section, the protocol was amended on March 14, 2025 (version 1.4), prior to restarting formal recruitment on June 1, 2025. One participant enrolled under the original protocol experienced intraoperative epidural leakage during TISI, meeting the definition of technical failure. In accordance with the principle that patients enrolled under a prior protocol version may be excluded from the primary analysis when the protocol has been substantially amended before formal analysis, this participant is not included in the modified intention-to-treat population. This decision was documented in the trial master file and approved by the trial steering committee. The participant will be reported separately as a pre-amendment pilot case in the final trial report.

#### 2.8.2 Primary and secondary outcomes analysis

Longitudinal repeated measures of primary and secondary outcomes will be analyzed using a linear mixed model. The model will include fixed effects for treatment group (TISI vs. TESI), time, the group-by-time interaction, and the respective baseline score as a covariate for adjustment. If clinically meaningful imbalances in key prognostic factors (e.g., age, sex, BMI, disease duration) are observed despite randomization, sensitivity analyses will be conducted with these variables included as additional fixed effects to assess the robustness of the treatment effect estimates. Least-squares means (LSMs) and 95% confidence intervals (CIs) will be calculated. The primary hypothesis test is the between-group comparison of the NRS at the 1-month primary endpoint. For all secondary outcomes at all post-baseline time points, between-group differences in LSMs will be reported. In addition to reporting least-squares means and 95% confidence intervals, we will present the proportion of responders (defined as achieving ≥MCID improvement) and the number needed to treat to aid clinical interpretation. To account for multiplicity in these secondary analyses, the false discovery rate will be controlled using the Benjamini-Hochberg procedure. Missing data will be handled using multiple imputation under the missing-at-random assumption, with predictive mean matching applied to continuous variables to generate 20 imputed datasets. Sensitivity analyses will be conducted to evaluate the robustness of the conclusions, including a complete-case analysis and/or an analysis under different missing data mechanisms.

#### 2.8.3 Exploratory mechanistic analyses

Exploratory analyses will examine whether longitudinal changes in radiological outcomes (Modic classification and Pfirrmann grade) are associated with changes in clinical outcomes over time. To explore potential mechanistic differences between interventions, interaction terms between treatment group and radiological changes will be evaluated to determine whether these associations differ by treatment arm. Given the ordinal nature of Modic classification and Pfirrmann grade, appropriate modeling strategies (e.g., generalized linear mixed models or ordinal regression) will be applied. These analyses are considered hypothesis-generating.

#### 2.8.4 Other analysis

For other between-group comparisons (e.g., baseline characteristics), continuous variables will be analyzed using independent-sample *t*-tests or Mann–Whitney *U* tests, as appropriate, and categorical variables will be compared using chi-square or Fisher’s exact tests.

All statistical tests will be two-sided, and a *p*-value <0.05 will be considered statistically significant. Data analysis will be performed using SAS software (version 9.4).

## 3 Discussion

The management of chronic DLBP with active discopathy remains a significant clinical challenge. While both TISI and TESI are employed in clinical practice for this condition, a direct, high-quality comparison of their efficacy and safety is lacking in the literature. This single-center, prospective, randomized controlled trial is designed to fill this critical evidence gap by providing a head-to-head comparison of TISI versus TESI.

The diagnosis of DLBP itself is complex and has been a subject of long-standing debate. MRI, while excellent for characterizing morphology, lacks sufficient specificity to definitively identify a degenerated disc as the source of pain (35, 36). Provocative discography, historically considered the reference standard, is invasive and controversial due to concerns about its false-positive rates and potential to accelerate disc degeneration (28, 35). In this trial, we have deliberately chosen not to use provocation discography as a diagnostic criterion. This decision is methodologically crucial for a direct comparison of TISI and TESI, as performing discography in the TESI group would introduce a confounding disc puncture, potentially biasing the results. Instead, we rely on a stringent, multi-component clinical and radiological definition of DLBP with active discopathy. This approach is grounded in both evolving expert recommendations and a body of literature that provides criteria for differential diagnosis, aiming to identify a specific disc-derived inflammatory pain phenotype and to distinguish it from other potential sources of low back pain (14, 15, 37–40). This non-invasive diagnostic approach aims to enhance the clinical applicability of our findings while avoiding the potential morbidity associated with discography.

The therapeutic landscape for DLBP is diverse but often characterized by limited and conflicting evidence (35, 41). Treatment options span a wide spectrum, from conservative management and multidisciplinary rehabilitation to various interventional procedures, progressing to surgical options and, more recently, emerging biological therapies. Among these, spinal injection procedures, particularly TESI and TISI, are widely adopted in clinical practice due to their technical accessibility and potential for immediate symptom relief (19, 25). The fundamental distinction between TESI and TISI lies in their anatomical target and proposed mechanism of action. TESI aims to suppress inflammation in the epidural space and around the nerve root, potentially affecting pain mediated by structures adjacent to the disc. TISI, however, delivers the corticosteroid directly into the nucleus pulposus, intending to quell the inflammatory cascade at its presumed source within the disc and adjacent Modic Type 1 endplates. Whether this more targeted intradiscal delivery yields clinically meaningful incremental benefit and/or greater durability compared with epidural delivery remains uncertain. Accordingly, our protocol frames the head-to-head comparison as both a pragmatic effectiveness question and a mechanistic test of the importance of delivery target. Participants will not be informed However, this potential benefit must be weighed against theoretical risks, including the possibility that disc puncture during TISI could, in the long term, contribute to accelerated disc degeneration (27, 28). Our trial is uniquely positioned to evaluate this risk–benefit ratio directly.

The selection of a 1-month post-intervention time point as the primary endpoint warrants careful justification, particularly in the context of a chronic pain condition. We acknowledge that sustained improvement over months to years is the ultimate therapeutic goal for chronic discogenic low back pain. However, the 1-month endpoint was chosen based on a combination of pharmacological, methodological, and pragmatic considerations. From a pharmacodynamic perspective, the anti-inflammatory and analgesic effects of corticosteroids typically peak within the first few weeks after injection; therefore, assessing outcomes at 1 month provides a valid and sensitive measure of the intervention’s biological activity. This approach is consistent with the highest-quality evidence in the field: landmark randomized trials of intradiscal glucocorticoid injection for active discopathy (9, 26) as well as a rigorous trial of epidural steroid delivery (30) have all prespecified 1-month or 4-week outcomes as primary efficacy endpoints. Adopting this common metric ensures comparability across studies and facilitates future evidence synthesis. Importantly, the 1-month primary endpoint is not intended to stand alone as the sole indicator of clinical value; rather, it serves as a confirmatory anchor within a comprehensive longitudinal framework. According to the ICH E9 (R1) estimand framework (42), the primary estimand targets the immediate treatment effect at the time of expected peak response, while the durability of benefit is assessed through repeated measures at 3, 6, and 12 months using linear mixed models. The sample size was powered to detect a minimal clinically important difference of 1.65 points on the NRS, ensuring that the primary comparison is grounded in clinical relevance. Ultimately, the totality of evidence—including pain trajectories, functional outcomes, quality of life, and structural changes over the full 12-month follow-up—will inform the clinical utility of TISI versus TESI in this patient population.

The management of chronic DLBP with active discopathy demands evidence-based strategies that are both effective and safe. The current equipoise between the targeted intradiscal approach (TISI) and the more conventional epidural approach (TESI) necessitates a direct comparative investigation. The findings from this trial will provide high-level evidence to inform clinical decision-making and guide the development of clinical practice guidelines. By systematically comparing the time course of pain relief, functional improvement, and structural changes over time, this study will contribute significantly to our understanding of the optimal interventional corticosteroid management for DLBP with active discopathy.

## Monitoring

An independent Data Monitoring Committee (DMC) comprising a spinal surgeon, pain specialist, and biostatistician will periodically review accumulated safety data. The DMC will operate under a dedicated charter, independent from sponsors and funders, and provide recommendations regarding trial continuation. No formal interim efficacy analyses are planned; however, the DMC may recommend early termination based on safety concerns, with exclusive access to unblinded interim results. Trial conduct will be monitored through regular site visits to verify protocol compliance and data accuracy.

## Dissemination policy

The trial results, whether positive, negative, or inconclusive, will be disseminated through peer-reviewed scientific journals and presentations at relevant national and international academic conferences. We also plan to disseminate a summary of the findings to the study participants and the public through appropriate channels, such as a lay-language summary on the hospital’s website.

## Protocol version

Protocol version 1.4, dated March 14, 2025. This version includes major amendments to the exclusion criteria (criteria 2: MRI evidence of a full-thickness annular fissure) and the addition of Pfirrmann grade as an exploratory radiological outcome. The full trial protocol (Version 1.4) is available upon reasonable request from the corresponding author.
